# High-efficiency, large-area, topology-optimized metasurfaces

**DOI:** 10.1038/s41377-019-0159-5

**Published:** 2019-05-29

**Authors:** Thaibao Phan, David Sell, Evan W. Wang, Sage Doshay, Kofi Edee, Jianji Yang, Jonathan A. Fan

**Affiliations:** 1 Department of Electrical Engineering, Stanford University, Stanford, CA 94305 USA; 2 Department of Applied Physics, Stanford University, Stanford, CA 94305 USA; 3 Université Clermont Auvergne, Institut Pascal, BP 10448, F-63000 Clermont-Ferrand, France; 4 CNRS, UMR 6602, Institut Pascal, F-63177 Aubière, France

**Keywords:** Metamaterials, Microresonators

## Abstract

Metasurfaces are ultrathin optical elements that are highly promising for constructing lightweight and compact optical systems. For their practical implementation, it is imperative to maximize the metasurface efficiency. Topology optimization provides a pathway for pushing the limits of metasurface efficiency; however, topology optimization methods have been limited to the design of microscale devices due to the extensive computational resources that are required. We introduce a new strategy for optimizing large-area metasurfaces in a computationally efficient manner. By stitching together individually optimized sections of the metasurface, we can reduce the computational complexity of the optimization from high-polynomial to linear. As a proof of concept, we design and experimentally demonstrate large-area, high-numerical-aperture silicon metasurface lenses with focusing efficiencies exceeding 90%. These concepts can be generalized to the design of multifunctional, broadband diffractive optical devices and will enable the implementation of large-area, high-performance metasurfaces in practical optical systems.

## Metasurfaces: Bigger and better ways to manipulate light

A new strategy for designing and manufacturing ‘metasurfaces’ - surfaces patterned at scales below the wavelength of light - will allow wider exploration of their optical effects. The sub-wavelength patterning of ultra-thin metasurfaces manipulates electromagnetic waves, including visible light, for use in many applications including sophisticated optical systems, sensing applications and optical computing. Researchers in the USA and France, led by Jonathan Fan at Stanford University, devised a novel three-step process to optimally design, manufacture and then “stitch together” small metasurface sections to create larger scale structures. Previous size limitations due to the extensive computational resources required to design the surfaces were overcome by the step that combines smaller sections with optimized topology. The team demonstrated the power of their procedures by making large area silicon metasurfaces that can focus light with impressively high efficiency.

## Introduction

**Fig. 1:**
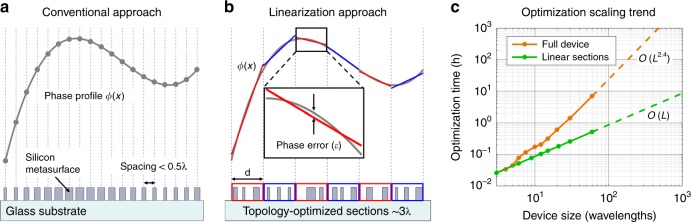
Strategies for metasurface design. **a** Conventional approaches sample the desired phase profile at discrete points and specify phase shifting elements to form a nanoscale phased array. **b** Our approach is to divide the desired phase profile into wavelength-scale, linear sections and use topology optimization to design each section individually. **c** Computation time versus device size for topology-optimized metasurfaces that are designed using two approaches: direct optimization of the entire metasurface (orange) and optimization of the metasurface after division into 3λ-wide sections (green)

Metasurfaces are optical devices that utilize subwavelength-scale structuring to shape and manipulate electromagnetic waves^1^. They are powerful complements to bulk refractive and scalar diffractive optics and have a wide range of potential applications in imaging^2^, lithography^3^, sensing^4^, and computing platforms^5^. To date, a broad range of metasurface design concepts have emerged. The most widely used methods, which we will term “conventional methods,” sample the desired phase profile using discrete phase-shifter elements to form a nanoscale phased array (Fig. 1a). These methods utilize a library of simple, physically intuitive building blocks, including anisotropic waveguides^6^, Mie resonators^7^, plasmonic resonators^8^, and dielectric transmit arrays^9^, and can quickly produce macroscale device designs. However, these approaches lack the necessary degrees of freedom for realizing high-efficiency in devices that are designed for large-angle deflections, multiple functions, and broadband responses^10^, thereby preventing metasurfaces from being practically applied in many contexts.

Topology optimization is an alternative design method that can overcome the efficiency limitations of conventional approaches^11^. Inverse design methods, including objective-first and adjoint-based topology optimization^12^, have produced photonic crystals^13,14^, optical demultiplexers^15^, and spectral splitters^16^ with outstanding performance resulting from the devices’ unusual geometries and nonintuitive optical dynamics. More recently, topology optimization has led to high-performance metasurfaces that have a broad range of capabilities, such as high-efficiency light deflection with nearly arbitrary input and output angles^17^, spectral sorting of plane waves to distinct diffraction orders^18^, and light focusing with field-curvature correction^19^. While devices that are designed using topology optimization support enhanced efficiencies, they require extensive computational resources that dramatically increase with the size of the device. Hence, current topology-optimized devices are either microscale in dimension or limited to periodic structures with microscale unit cells.

We report a conceptually new approach to metasurface design that extends the high performance of topology-optimized devices to macroscopic areas in a computationally efficient manner. The concept is schematically illustrated in Fig. 1b and is comprised of three parts. First, we discretize the desired phase profile into a series of wavelength-scale, linear sections. Next, we use topology optimization to design metasurface elements that optimally scatter light as required by each section of the phase profile. Finally, we stitch all the elements together to form a full metasurface. Our approach produces devices that operate more efficiently than conventional designs by accounting for and optimizing the near-field optical coupling between neighboring nanostructures. Our design method is also substantially more computationally efficient than existing topology optimization methods.

## Results

### Assessment of computational efficiency

To benchmark the improvements in computational efficiency that are afforded by our approach, we perform adjoint-based topology optimization on metagratings that are made of silicon ridges. These periodic metasurfaces are designed to deflect light of a specified wavelength λ to the +1 diffraction order. We perform electromagnetic simulations for topology optimization using rigorous coupled wave analysis (RCWA), which is also known as the Fourier modal method^20–23^, on a personal computer and plot the time that is required for optimizing a full device as a function of its width *L* in Fig. 1c. The simulation time scales approximately as ${\cal{O}}\left( {L^{2.4}} \right)$, which corresponds to the general scaling trend for electromagnetic solvers that utilize standard matrix multiplication and inversion algorithms^24^. These trends demonstrate that prohibitively large computational resources are required for directly optimizing devices that are many times larger than the wavelength.

If we instead divide the metagrating into sections of width *d*, we have *L*/*d* sections to optimize. The total computation time now scales as ${\cal{O}}\left( {d^{2.4} \cdot L/d} \right) = {\cal{O}}\left( {d^{1.4} \cdot L} \right)$, which is a linear function of the total size. This expression also shows that the computation time decreases with decreasing section size. In practice, we found that using 3λ-wide sections minimizes the total computation time, as the benefits from using smaller sections are outweighed by the computational overhead in the electromagnetic solver. We plot the computation time for a metasurface that has been optimized using 3λ-wide sections in Fig. 1c. The observed reduction in the computation time enables us to optimize devices with dimensions that are many times larger than the wavelength using realistic computational resources. For example, via our method, we can produce millimeter-scale topology-optimized metasurfaces that operate at near-infrared wavelengths in less than 1 day using a personal computer. In contrast, optimizing the full device at once would take nearly 1 year and require intractably large amounts of memory.

We realize additional savings in computation time via our method when using multiple computing cores because individual segments can be optimized on different cores. Our design concept allows the segments to be treated independently, thereby enabling the optimizations to be parallelized without issues concerning race conditions and synchronization^25^. With *N* computing nodes, metasurfaces that are subdivided into *N* sections can be optimized in the same amount of time as it takes to optimize a single segment.

These reductions in computational complexity also apply to fully three-dimensional topology-optimized metasurfaces, which exhibit even more severe scaling trends: the time it takes to optimize a metasurface of size *L* × *L* all at once scales approximately as ${\cal{O}}\left( {L^{4.8}} \right)$. This trend indicates the necessity of linear sectioning for these more intricate design problems, which would reduce the runtime to a more reasonable ${\cal{O}}\left( {L^2} \right)$.

### Sectioning of a curvilinear phase profile

The process of approximating a curvilinear phase profile with a series of linear sections introduces wavefront error, which is denoted as ϵ (Fig. 1b, inset). However, this error has a negligible impact on the overall metasurface performance if the sections are sufficiently small. To analyze the effect of linearizing a general curvilinear phase profile *ϕ*(*x*), we locally describe each section of *ϕ*(*x*) at location *x*_0_ using a 2^nd^-order Taylor series expansion as follows:

1 $$\phi \left( x \right) \approx \phi \left( {x_0} \right) + \phi \prime \left( {x_0} \right)\left( {x - x_0} \right) + \frac{1}{2}\phi \prime \prime \left( {x_0} \right)\left( {x - x_0} \right)^2$$

This section can be approximated as a line of slope $\phi \prime \left( {x_0} \right)$ with a phase offset of *ϕ*(*x*_0_) + Δ*ϕ*, which incurs an error of ${\it{\epsilon }}\left( x \right) = \phi \prime \prime \left( {x_0} \right)\left( {x - x_0} \right)^2/2 - {\mathrm{\Delta }}\phi$. Given a section of length $d$ at position *x*_0_, the root-mean-square (RMS) wavefront error is minimized when ${\mathrm{\Delta }}\phi = \phi \prime \prime \left( {x_0} \right)d^2/24$ and is expressed as follows:

2 $${\it{\epsilon }}_{{\mathrm{rms}}}\left( {{\mathrm{\Delta }}x} \right) = \frac{1}{{12\sqrt 5 }}\phi \prime \prime \left( {x_0} \right)d^2$$

We use this result to analyze the impact of linearization on a focusing cylindrical lens, which enables us to benchmark the device performance using well-established metrics in lens design. To quantify the performance of lenses that are constructed with linear sections, we use the Strehl ratio^26^, which is a metric that compares the diffraction efficiency of our lenses to that of an ideal lens. For a lens that focuses normally incident light, the ideal phase profile is $\phi \left( x \right) = \left( {2\pi /\lambda } \right)( {f - \sqrt {f^2 + x^2} } )$^27^, where *f* is the focal length and *λ* is the wavelength. A lens with a Strehl ratio of 0.98, which corresponds to an RMS wavefront error of λ/50, can be realized if we use linear sections that are no larger than the following:

3 $$d\, < \,0.73\sqrt {f\lambda }$$

**Fig. 2:**
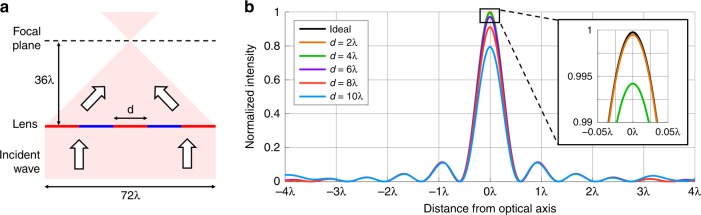
Impact of phase profile linearization on the cylindrical lens performance. **a** Cylindrical lenses of focal length 36λ and NA of 0.7 are constructed using linear sections of length *d* for various values of *d*. **b** Line scans of the field intensity at the focal planes of the lenses. Lenses that are linearized with sections that are smaller than 4λ have peak intensities that are within 1% of that of the ideal lens

This equation provides a practical and quantitative guide for linearizing phase profiles in a manner that minimizes the phase error. As an example, consider a cylindrical lens with a focal length of *f* = 36*λ* and a numerical aperture (NA) of 0.7 (Fig. 2a). Using Eq. 3, we expect that linearizing the phase profile with segments that are smaller than 4.4λ will have a negligible impact on the performance. To verify, we simulate lenses that are linearized with various segment lengths and calculate the field intensities at the focal plane. These field intensity profiles are plotted in Fig. 2b, according to which the lenses that are linearized with section lengths of 2λ and 4λ are nearly indistinguishable from the ideal lens.

Our approach to sectioning readily extends to three-dimensional phase profiles, which can be approximated as series of planar tiles. This approximation is discussed in detail in the Supplementary Section. For a hyperboloid that is discretized into tiles with dimensions *d* × *d*, an RMS wavefront error of λ/50 can be realized if:

4 $$d\, < \,0.61\sqrt {f\lambda }$$

### Topology optimization for finite-sized, isolated devices

To design metasurface elements that have the desired linear phase profiles, we utilize adjoint-based topology optimization^28^. Adjoint-based optimization is an iterative algorithm that modifies the device’s dielectric constant distribution, namely, *ε*(*x*), to maximize a figure of merit (FoM). Our objective is to optimize a device that scatters normally incident electromagnetic waves in a desired direction with electric field amplitude ***E***_tgt_ and phase *ϕ*_tgt_. To compute the FoM, we run a forward simulation in which waves that are incident onto the metasurface element scatter in the desired direction with field amplitude ***E***_fwd_ and phase *ϕ*_fwd_. Near-to-far-field transformations from the forward simulations are used to evaluate ***E***_fwd_ and *ϕ*_fwd_^29^. The FoM describes the difference between the current and desired responses and has the following form:

5 $$FoM = - A_1\left[ {\left| {\boldsymbol{E}}_{\text{tgt}} \right|^2 - \left| {\boldsymbol{E}}_{\text{fwd}} \right|^2} \right]^2 - A_2\left[ {\arg \left( {e^{i\left( {\phi _{\text{tgt}} - \phi _{\text{fwd}}} \right)}} \right)} \right]^2$$

The terms *A*_1_ and *A*_2_ are weights that balance how strongly the FoM is biased toward optimizing the amplitude and phase, respectively. To determine how *ε*(*x*) should be modified to improve the FoM each iteration, we perform a pair of forward and adjoint simulations and record the electric fields in the device for each excitation condition. These fields are used to calculate *δFoM*, which is the gradient of the FoM with respect to the dielectric constant at each position *x*:

6 $$\begin{array}{l}\delta FoM = {2}A_{1}\left( {\left| {\boldsymbol{E}}_{\text{tgt}} \right|^{2} - \left| {\boldsymbol{E}}_{\text{fwd}} \right|^2} \right){\cal{R}}e\left\{ {{\boldsymbol{E}}_{\text{fwd}} \cdot \delta {\boldsymbol{E}}^\ast } \right\}\\ - {2}A_{2}\left( \phi _{\text{tgt}} - \phi _{\text{fwd}} \right)\frac{1}{{\left| {\boldsymbol{E}}_{\text{fwd}} \right|^2}}{\cal{I}}m\left\{ {\boldsymbol{E}}_{\text{fwd}} \cdot \delta {\boldsymbol{E}^ \ast } \right\}\end{array}$$

where $\delta {\boldsymbol{E}}$ is a function of the adjoint field and represents the variations of the field in the target direction in response to variations of the refractive index within the device. A more detailed discussion of the adjoint optimization method that is applied here is provided in the Supplementary Section.

### Aperiodic Fourier modal method

To apply these concepts to the design of isolated, finite-sized device elements, we have developed an aperiodic Fourier modal method (AFMM), which is a hybrid method that combines a solver for periodic systems with perfectly matched layers (PMLs). The key challenge of implementing PMLs involves describing both the periodic incident plane wave (the input field) and the aperiodic scattered field (the output field) of the isolated device within the same formalism. To address this challenge, we introduce a hybrid method that combines a Fourier basis, Maxwell’s equations in complex coordinates, and the Stratton-Chu integral formalism^30–34^.

A metasurface is typically composed of a single layer of patterned material. The patterned material can be expressed as a distribution of the relative permeability, namely, *ϵ*(*x, y*), and the permeability, namely, *μ*(*x, y*), on the *xy*-plane. Along the thickness of the device in the *z*-direction, the device cross-section is constant. In this case, it can be shown from Maxwell’s equations that the transverse electric fields satisfy the following eigenvalue equation:

7 $$- \gamma ^2\left[ {\begin{array}{*{20}{c}} {{\boldsymbol{E}}_x} \\ {{\boldsymbol{E}}_y} \end{array}} \right] = {\cal{L}}_{EH}{\cal{L}}_{HE}\left[ {\begin{array}{*{20}{c}} {{\boldsymbol{E}}_x} \\ {{\boldsymbol{E}}_y} \end{array}} \right]$$

where ${\cal{L}}_{EH}$ and ${\cal{L}}_{HE}$ are differential operators that are based on *ε* and *μ* and the electric field solution can be expressed as ${\mathrm{\Phi }}\left( {x,y} \right)e^{ - ik\gamma z}$. The Fourier modal method can easily be used to expand the input fields, which are assumed to be periodic, into a Fourier basis:

8 $${\mathrm{\Phi }}_{in}\left( {x,y,z} \right) = \mathop {\sum }\limits_p A_pe^{ - ik\gamma _pz}\mathop {\sum }\limits_{nm} \delta _{nm,p}e^{ - ik\alpha _nx}e^{ - ik\beta _my}$$

Next, the PML boundary conditions are described similarly. The PMLs can be introduced via a change of coordinates $\left( {x,y,z} \right) \to (\tilde x,\tilde y,\tilde z)$^30,31^:

9 $$\left\{ {\begin{array}{*{20}{c}} {\tilde x\left( x \right) = \left( {\chi _x - i\eta _x} \right)\left( {x - x_0} \right) + x_0} \\ {\tilde y\left( y \right) = \left( {\chi _y - i\eta _y} \right)\left( {y - y_0} \right) + y_0} \end{array}} \right\}$$

Here, the parameter *χ* controls the scaling of the PML layers, while the parameter *η* controls the PML attenuation. This transformation is also useful for expressing the output scattered fields, which are computed as eigenfunctions of Maxwell’s equations in complex coordinates:

10 $${\mathrm{\Phi }}_s\left( {x,y,z} \right) = \mathop {\sum }\limits_p B_pe^{ - ik\gamma _pz}\mathop {\sum }\limits_{nm} {\mathrm{\Phi }}_{nm,p}e^{ - ik\alpha _nx}e^{ - ik\beta _my}$$

Finally, the Stratton-Chu integral equation computes the radiated field in all of space^34^. In this study, we design devices that are comprised of nanoridges, which can be described in this analysis by a one-dimensional Fourier basis.

### Section optimization demonstration

**Fig. 3:**
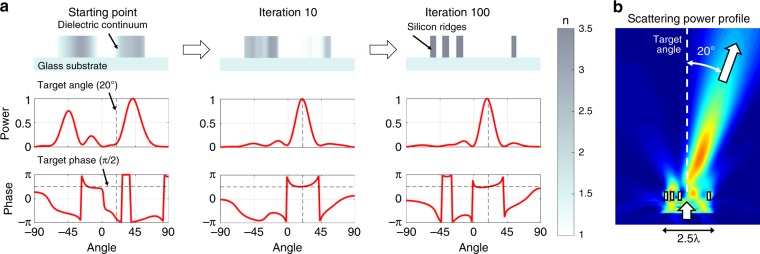
Metasurface element optimization. **a** At the beginning of the optimization process, the initial dielectric distribution is a random dielectric continuum. The dashed lines indicate the desired scattering angle of 20° and phase of π/2. After 10 iterations, the scattering profile is already highly directional. After 100 iterations, the optimization is complete and the metasurface section is a binary structure of silicon and air that supports the desired scattering metrics. **b** An intensity plot that shows the scattered fields of the optimized metasurface element

To demonstrate adjoint-based optimization in the design of metasurface elements, we design a 2.5λ-wide element that scatters incident TM-polarized light at a 20° angle with a phase response of π/2. The dielectric distribution and the amplitude and phase scattering profiles of the element at successive iterations in the optimization process are shown in Fig. 3a. The optimization begins with a random dielectric continuum with values between air and silicon. After a few iterations, the continuum begins to strongly scatter light at the desired angle. The final metasurface section is a binary structure of silicon in air that possesses a peak scattering amplitude and phase response matching with the targeted values. The full width at half maximum of the scattering peak is consistent with that expected from light that is diffracting from a 2.5λ-wide aperture; hence, the element is performing directional scattering near its physical limits.

An intensity plot of the scattered fields from the fully optimized section (Fig. 3b) shows strong near-field coupling between neighboring nanostructures; hence, optimal near-field coupling is responsible for mediating strong scattering in the desired direction. As a method of gradient descent, adjoint-based topology optimization is a local optimizer and is sensitive to the initial dielectric distribution^10^. To obtain high-performance elements for a desired scattering angle and phase target, we perform ten optimizations with various initial dielectric distributions and select the best result.

### Optimized metalens simulation results

Combining everything into a proof-of-concept demonstration, we stitch together optimized metasurface elements to construct cylindrical metalenses. We design these metalenses to focus TM-polarized light at a wavelength of 640 nm. To enable device operation at visible wavelengths, we use 250-nm-thick crystalline silicon, which has relatively low absorption compared to polycrystalline and amorphous silicon, but much higher index contrast than materials such as titanium dioxide^35,36^.

First, we design and simulate 64-μm-wide metalenses with NAs that range from 0.2 to 0.9. We divide the metalenses into sections that are 2 μm wide, which is below the phase error limit of Eq. 3 and near the optimal size for efficient computation (Fig. 1c). Further reductions in section size lead to degradation of the device performance. The reason can be traced to our design of each metasurface element, which is optimized in isolation with PML boundary conditions. When the elements are stitched together to produce a device, the optical fields that are guided by a single element have evanescent tails and can couple to a neighboring element in a parasitic manner. Smaller section sizes require more elements to be stitched together to produce a desired metasurface, thereby resulting in more boundaries and more parasitic coupling. Below a section size of 2λ, the device performance begins to degrade and below a section size of 1λ, the aperiodic boundary conditions in our optimizer are no longer valid.

There are a few approaches for addressing the issue of stitching error. One is to keep the section size relatively large compared to the wavelength. Another is to perform boundary optimization on the stitched regions to eliminate the stitching error. A third approach, which we use here, is to separate silicon structures from other sections by a gap of at least 0.2λ, thereby reducing the near-field coupling between sections. To ensure a reduction in the stitching error with this scheme, we simulate stitched sections to check for spurious diffraction and redesign the sections in the event of excess error.

**Fig. 4:**
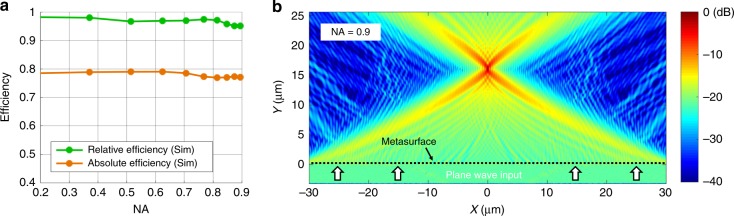
Simulation results of optimized metalenses. **a** Relative and absolute efficiencies for metalenses that were designed with various numerical apertures. **b** A full-field simulation of a metalens with an NA of 0.9

The efficiencies of our simulated metalenses are summarized in Fig. 4a. The absolute efficiency is defined as the amount of power that is contained in the principal lobe of the focus compared to that of an ideal lens with 100% transmission. The relative efficiency, or focusing efficiency, compares the power in the principal lobe to that of an ideal lens that transmits the same amount of power as the device^37^. This efficiency corresponds to the efficiency of the diffraction process, as it removes the effects of absorption from the material and reflection at the metalens interface.

The efficiency plots show that the relative efficiencies are consistently high, namely, above 93%, with minimal drop-off in performance as the NA increases. This trend is unlike that of conventional metalenses, where the efficiency decreases with increasing NA because conventional designs cannot efficiently deflect light at large angles^27,38^. The absolute efficiencies of the metalenses all exceed 75%, with approximately 10% of the light reflected from the metalens and 10% absorbed by the silicon. Reflection losses can be reduced via the use of more intricate three-dimensional silicon nanostructures, while absorption losses can be minimized by designing silicon-based devices for longer wavelength operation^28^. A simulated field profile of a metalens with an NA of 0.9 is shown in Fig. 4b, which demonstrates that the lens focuses strongly with minimal spurious diffraction.

### Performance of fabricated metalenses

We design, fabricate, and characterize 200-μm-wide metalenses with NAs of 0.2, 0.5, and 0.8. To prepare crystalline silicon thin films on glass, we use hydrogen silsesquioxane to bond silicon-on-insulator wafers onto Pyrex wafers under high temperature and pressure^36,39^. After removing the silicon handle wafer and the buried oxide layer, we pattern and etch the devices via standard electron beam lithography and dry etching techniques. We characterize the metalenses by collimating polarized, monochromatic light from a tunable white-light laser onto the devices and imaging the light at the focal plane with a ×100 objective (NA = 0.9) and a CCD sensor.

**Fig. 5:**
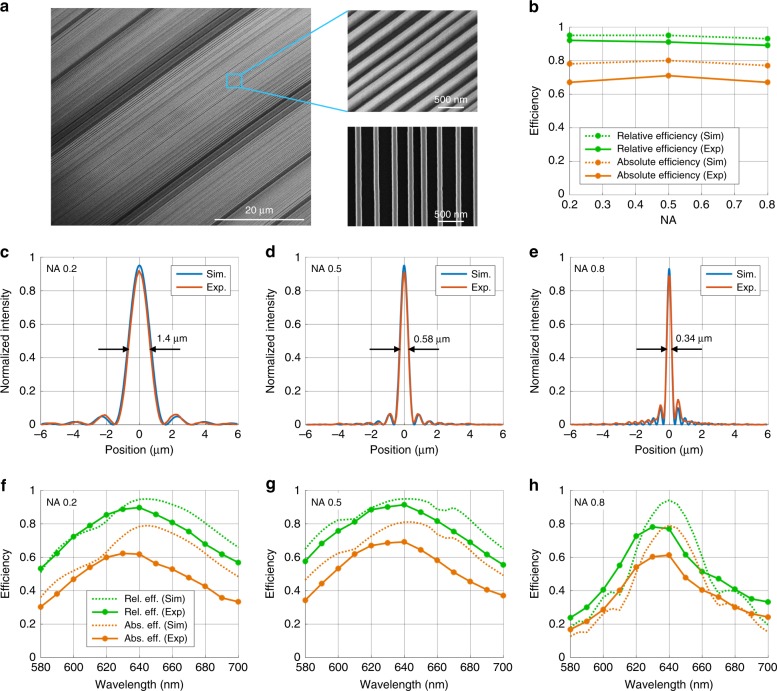
Experimental characterization of fabricated metalenses. **a** Scanning electron micrographs of the metalenses with tilted and top-down views. **b** Relative and absolute efficiencies of the fabricated metalenses, along with their simulated values. **c**–**e** Intensity line scans at the focal planes of the metalenses with NAs of 0.2, 0.5, and 0.8, respectively, along with comparisons with the simulated lenses. **f**–**h** Efficiencies of the fabricated metalenses as a function of the wavelength, along with their simulated values. These metalenses have the same design but were fabricated on a different sample than those that were measured in (**a**–**e**)

Scanning electron microscope images of the center of a representative device are shown in Fig. 5a and show silicon nanostructures that exhibit smooth and vertical sidewalls. The metalenses all have relative efficiencies that exceed 89% and absolute efficiencies that exceed 67% for all NAs, which are within 10% of the simulated values (Fig. 5b). All the metalenses exhibit diffraction-limited performance, as shown by the theoretical and experimental intensity plots in Fig. 5c–e. The device with an NA of 0.8 can focus light at a wavelength of 640 nm to a spot with a beam waist of 340 nm. The central lobes of the foci are all much stronger than the side lobes; hence, the focusing efficiency is high.

The metalenses maintain reasonably high efficiencies for wavelengths that range from 580 to 700 nm, as shown in Fig. 5f–h. As these lenses are not designed to be achromatic, the focal length shifts with the wavelength. The simulated shifts are shown in the Supplementary section in Fig. S3. Future work will focus on generalizing our design approach to include achromatic functionality, which can be addressed by modifying the optimizer’s figure of merit to include multiple wavelengths^18^. The figure of merit can be specified so that each metasurface section deflects all wavelengths in the same direction and realizes the correct dispersion for ensuring constructive interference at the focus^40,41^.

## Discussion

In summary, we present a computationally efficient method for designing large-area, topology-optimized metasurfaces. Our approach of approximating a desired phase profile with a series of linear segments renders the design problem computationally tractable while minimally impacting the device performance. Wavelength-scale scattering elements that are designed via topology optimization can strongly scatter light at a desired angle and phase, thereby serving as high-performance building blocks for larger metasurfaces.

The metalenses that are demonstrated here have limited functionality and other heuristic design methods are available that can produce high-performance, high-numerical-aperture metalenses^42^. What differentiates our method is that our design platform can be generalized systematically to multifunctional, broadband, and even multilayer metasurfaces with large areas and high efficiencies. To practically extend topology optimization in these directions, continued improvements in electromagnetic solver speed will be required, particularly for fully three-dimensional devices. For multilayer devices, new innovations will be needed to address the stitching error, which can compound due to interactions between neighboring elements. We envision that these design methods will enable the realization of compact and monolithic electromagnetic devices that exhibit high efficiencies and practical functionality across the electromagnetic spectrum.

## Materials and methods

### Sample fabrication

Crystalline silicon on glass wafers was prepared via an approach that is similar to that done by Sell, et al.^36^. A Pyrex and silicon-on-insulator (SOI) wafer were bonded under high temperature and pressure using hydrogen silsesquioxane. The silicon handle wafer was etched away using SF_6_ plasma and the buried oxide layer was etched away using HF solution. The wafer was diced into pieces for later use. The metasurfaces were patterned onto a piece in the AR-P-6200 resist via electron beam lithography. Aluminum oxide was evaporated onto a piece and removed using liftoff to form a hard mask. The silicon was etched using a Cl_2_ and HBr plasma. The aluminum oxide was removed using a solution of HCl at 50 °C. Additional details can be found in the supplementary information.

### Measurement procedure

A collimated beam of light from a tunable white light laser is filtered with a longpass filter and polarized with a linear polarizer. Then, the beam passes through a weak cylindrical lens to focus it onto the metasurface aperture without significantly changing the angle of incidence. The metasurface focuses the light. Next, the focal plane is imaged onto a CMOS detector using a 0.9 NA, 100x objective and a tube lens.

To evaluate the lens efficiencies, a line scan of the focal plane image is obtained. The lens relative efficiency is calculated by integrating over a small aperture around the main focal lobe and comparing it to what would be expected for an ideal lens of the same numerical aperture. The lens absolute efficiency is calculated by multiplying the relative efficiency by the measured transmission. A schematic diagram of the optical setup and additional characterization details can be found in the supplementary information.

### Simulations

We used Reticolo, a rigorous coupled-wave analysis solver, as a basis to perform optimizations^21^. The final metasurface lens designs were simulated using Lumerical FDTD. The efficiencies were calculated by determining the optical power that is contained in the main lobe of the focus and comparing it to that expected from an ideal lens.

## Supplementary information

Supplementary Information.
