# Supplementary material for: High-efficiency, large-area, topology-optimized metasurfaces
Source: Light Sci Appl. 2019 May 29;8:48. doi: 10.1038/s41377-019-0159-5 (PMC6538635; doi:10.1038/s41377-019-0159-5)
Supplement: Supplementary file 1 — Supplementary Information. [file 41377_2019_159_MOESM1_ESM.docx]

**Supplementary Section to: High-efficiency, large-area, topology-optimized metasurfaces**

Thaibao Phan^1^, David Sell^2^, Evan W. Wang^1^, Sage Doshay^2^, Kofi Edee^3^, Jianji Yang^1^, and Jonathan A. Fan^1*^

1. Department of Electrical Engineering, Stanford University, Stanford, CA 94305
2. Department of Applied Physics, Stanford University, Stanford, CA 94305
3. Université Clermont Auvergne, Institut Pascal, BP 10448, F-63000 Clermont-Ferrand, France, CNRS, UMR 6602, Institut Pascal, F-63177 Aubière, France

*Address correspondence to: [jonfan@stanford.edu](mailto:jonfan@stanford.edu)

**Contents:**

1. Derivation of Linearization Phase Error (Eqn. 3)
2. Calculation of Intensity and Phase Gradient for Performing Topology Optimization
3. Fabrication Details
4. Experimental Setup and Characterization Details
5. Linearization of 3D Metasurfaces
6. Discussion on Optimization and Scaling of 3D Metasurfaces
7. Outline and Flow Chart of Design Algorithm

Part 1: Derivation of Linearization Phase Error (Eqn. 3)

Consider a quadratic function $y_{1}\left( x \right)$ and a line $y_{2}\left( x \right)$ used to approximate it:

|  | $y_{1}\left( x \right)=ax^{2}+bx+c_{1}$ | (1.1) |
| --- | --- | --- |

|  | $y_{2}\left( x \right)=bx+c_{2}$ | (1.2) |
| --- | --- | --- |

The difference $\epsilon\left( x \right)$ between the two functions is:

|  | $\epsilon\left( x \right)=y_{1}\left( x \right)-y_{2}\left( x \right)=ax^{2}+c_{3}$ | (1.3) |
| --- | --- | --- |

Where $c_{3}=c_{1}-c_{2}$. Over a segment of length $d$ centered around 0, the mean square of the difference is:

$$\epsilon_{ms}\left( \pm d/2 \right)=\frac{1}{d}\int_{-d/2}^{d/2} \left( ax^{2}+c_{3} \right)^{2}dx$$

$$=\frac{2}{d}\int_{0}^{d/2} \left( a^{2}x^{4}+2ac_{3}x^{2}+c_{3}^{2} \right)dx$$

$$=\frac{2}{d}\left( \frac{a^{2}}{5}\frac{d^{5}}{32}+\frac{2ac_{3}}{3}\frac{d^{3}}{8}+c_{3}^{2}\frac{d}{2} \right)$$

|  | $\epsilon_{ms}=c_{3}^{2}+\frac{ad^{2}}{6}c_{3}+\frac{a^{2}d^{4}}{80}$ | (1.4) |
| --- | --- | --- |

The mean square error is minimized when its derivative with respect to the constant offset $c_{3}$ is zero:

|  | $\frac{d\epsilon_{ms}}{dc_{3}}=2c_{3}+\frac{ad^{2}}{6}=0$ | (1.5) |
| --- | --- | --- |

Therefore,

|  | $c_{3}=-\frac{ad^{2}}{12}$ | (1.6) |
| --- | --- | --- |

Substituting this value, we can explicitly calculate the mean square error as a function of $d$.

$$\epsilon_{ms}=\frac{a^{2}d^{4}}{144}-\frac{ad^{2}}{6}\frac{ad^{2}}{12}+\frac{a^{2}d^{4}}{80}$$

$$=a^{2}d^{4}\left( \frac{1}{144}-\frac{1}{72}+\frac{1}{80} \right)$$

|  | $\epsilon_{ms}=\frac{a^{2}d^{4}}{180}$ | (1.7) |
| --- | --- | --- |

Therefore, the root-mean-square difference is:

|  | $\epsilon_{rms}=\frac{\left\vert a \right\vert d^{2}}{6\sqrt{5}}$ | (1.8) |
| --- | --- | --- |

We can calculate the span in relation to a desired difference:

|  | $d=\sqrt{\frac{6\sqrt{5}}{\left\vert a \right\vert}\epsilon_{rms}}$ | (1.9) |
| --- | --- | --- |

In reference to the main text, we substitute the value $a=\phi''(x_{0})/2$ into (6) and find that the optimal offset for minimum mean square phase error is:

|  | $\Delta\phi=-\frac{\phi^{''}\left( x_{0} \right)d^{2}}{24}$ | (1.10) |
| --- | --- | --- |

An ideal focusing lens with a focal length $f$ at a wavelength $\lambda$ has a phase profile of:

|  | $\phi\left( x \right)=\frac{2\pi}{\lambda}\left( f-\sqrt{f^{2}+x^{2}} \right)$ | (1.11) |
| --- | --- | --- |

Its derivatives are:

|  | $\phi^{'}\left( x \right)=-\frac{2\pi}{\lambda}\frac{x}{\sqrt{f^{2}+x^{2}}}$ | (1.12) |
| --- | --- | --- |

|  | $\phi^{''}\left( x \right)=-\frac{2\pi}{\lambda}\frac{f^{2}}{{\sqrt{f^{2}+x^{2}}}^{3}}$ | (1.13) |
| --- | --- | --- |

The maximum value of $\phi^{''}$ occurs when $x=0$ and is equal to:

|  | $\phi^{''}\left( 0 \right)=-\frac{2\pi}{f\lambda}$ | (1.14) |
| --- | --- | --- |

Substituting into (9), we find that the span $d$ and phase error $\epsilon_{rms}$ are related by:

|  | $d=\sqrt{12\sqrt{5}\frac{\epsilon_{rms}}{2\pi}f\lambda}$ | (1.15) |
| --- | --- | --- |

For a maximum $\lambda/50$ RMS phase error, we let $\epsilon_{rms}<2\pi/50$, resulting in the constraint:

|  | $d<0.73\sqrt{f\lambda}$ | (1.16) |
| --- | --- | --- |

Part 2: Calculation of Intensity and Phase Gradient for Performing Topology Optimization

The complex field at the target point can be described by $\boldsymbol{E}\left( \boldsymbol{r}_{0} \right)=\left| E \right|e^{i\phi}\hat{\boldsymbol{e}}$**,** where $\hat{\boldsymbol{e}}$ is the polarization vector (assumed linearly polarized), $\boldsymbol{r}_{0}$ is an arbitrary target location in the far field, $E$ is the complex scalar field amplitude at $\boldsymbol{r}_{0}$, and $\phi=\arg\left\{ E \right\}$ is the phase of the field at $\boldsymbol{r}_{0}$.

**Intensity Figure of Merit**

We wish to optimize the field intensity $\left| E \right|^{2}$ at a target location in the far field. We define our intensity figure or merit (FoM) to be:

|  | $FoM_{I}=\left\vert E \right\vert^{2}$ | (2.1) |
| --- | --- | --- |

Under a perturbation to the field $\delta E$, we can calculate a corresponding perturbation to the FoM:

|  | $\delta FoM_{I}=\left\vert E+\delta E \right\vert^{2}-\left\vert E \right\vert^{2}=\left( E+\delta E \right)\cdot\left( E^{*}+\delta E^{*} \right)-E\cdot E^{*}\approx2\mathrm{Re}\{E^{*}\cdot\delta E\}$ | (2.2) |
| --- | --- | --- |

This expression is consistent with those previously used for optimizing metagrating beam deflection to a specific diffraction order (see, for example, Ref. [25]).

**Phase Figure of Merit**

The phase in terms of the field amplitude is:

|  | $\phi(E,E^{*})=\arctan\left[ \frac{\mathrm{Im}\left\{ E \right\}}{\mathrm{Re}\left\{ E \right\}} \right]=\arctan\left[ \frac{E-E^{*}}{E+E^{*}} \right]$ | (2.3) |
| --- | --- | --- |

A variation in the phase, resulting from a variation in field, $\delta E$, can be similarly evaluated:

|  | $\delta\phi\left( E,E^{*} \right)=\phi\left( E+\delta E,E^{*}+\delta E^{*} \right)-\phi\left( E,E^{*} \right)=\arctan\left[ f\left( E+\delta E,E^{*}+\delta E^{*} \right) \right]-\arctan\left[ f\left( E,E^{*} \right) \right]$ | (2.4) |
| --- | --- | --- |

Where:

|  | $f\left( E,E^{*} \right)\equiv\frac{E-E^{*}}{E+E^{*}}$ | (2.5) |
| --- | --- | --- |

Keeping only terms first order in $\delta E$, $\delta\phi$ becomes:

|  | $\delta\phi\approx\frac{1}{f\left( E,E^{*} \right)+1}\left[ \delta E\frac{\partial f}{\partial\left( \delta E \right)}+\delta E^{*}\frac{\partial f}{\partial\left( \delta E^{*} \right)} \right]=\frac{2}{\left\vert E \right\vert^{2}}\mathrm{Im}\{E^{*}\cdot\delta E\}$ | (2.6) |
| --- | --- | --- |

We define the FoM as the negative of the distance from a target phase:

|  | $FoM_{\phi}=-\left[ \arg\left( e^{i\left( \phi_{\mathrm{tgt}}-\phi\right)} \right) \right]^{2}$ | (2.7) |
| --- | --- | --- |

We can reframe the FoM to be:

|  | $FoM_{\phi}=-\left( \phi_{\mathrm{tgt}}-\phi\right)^{2}$ | (2.8) |
| --- | --- | --- |

The variation of the FoM can be expressed as:

|  | $\delta FoM_{\phi}=\left( \phi_{\mathrm{tgt}}-\phi\right)\delta\phi=\left( \phi_{\mathrm{tgt}}-\phi\right)\frac{2}{\left\vert E \right\vert^{2}}\mathrm{Im}\{E^{*}\cdot\delta E\}$ | (2.9) |
| --- | --- | --- |

**Evaluating** $\boldsymbol{\delta E}$

The gradient for the optimization requires that the variation of the FoM be expressed in terms of a variation in the design variables. The design variable in this case is the dielectric constant within the designated design domain $\mathcal{D}$, written as $\varepsilon(\boldsymbol{r})$ for $\boldsymbol{r}\mathcal{\in D}$.

Consider an arbitrary point in the design domain $\boldsymbol{r}_{i}$ which is subject to an electric field of $\boldsymbol{E}(\boldsymbol{r}_{i}\boldsymbol{)}$ when the current design is illuminated in the same way that it would be tested experimentally. If the dielectric constant at $\boldsymbol{r}_{i}$ were to be altered slightly such that $\varepsilon\left( \boldsymbol{r}_{i} \right)\boldsymbol{\to}\varepsilon\left( \boldsymbol{r}_{i} \right)+\delta\varepsilon$, we can make the approximation that the electric field at that point will not change significantly. Using this approximation, we can evaluate the effect of $\delta\varepsilon$ on the overall electric field by treating it as an induced polarization density over a volume $\Delta V$ centered at the location $\boldsymbol{r}_{i}$:

|  | $\boldsymbol{P}\left( \boldsymbol{r}_{i} \right)\sim\varepsilon_{0}\delta\varepsilon\Delta V\boldsymbol{E}\left( \boldsymbol{r}_{i} \right)$ | (2.10) |
| --- | --- | --- |

We can extend this so a small change in dielectric constant which affects the entire design domain, $\delta\varepsilon(\boldsymbol{r}\mathcal{\in D)}$. Using a Green’s function formalism, we can express the change to the field at the target point as:

|  | $\delta\boldsymbol{E}\left( \boldsymbol{r}_{0} \right)=\varepsilon_{0}\int_{\boldsymbol{r}^{\boldsymbol{'}}\mathcal{\in D}} \delta\varepsilon\left( \boldsymbol{r}^{'} \right)G\left( \boldsymbol{r}_{0},\boldsymbol{r}^{'} \right)\cdot\boldsymbol{E}\left( \boldsymbol{r}^{'} \right)d\boldsymbol{r}^{'}$ | (2.11) |
| --- | --- | --- |

Here, $G\left( \boldsymbol{r}_{0},\boldsymbol{r}^{'} \right)\cdot\boldsymbol{E}\left( \boldsymbol{r}^{'} \right)$ represents the field at location $\boldsymbol{r}_{0}$ induced by a dipole at location $\boldsymbol{r}^{'}$.

Within both (2.2) and (2.9), the term $\delta E$ appears in an expression containing $E^{*}\cdot\delta E$ or its complex conjugate. If that expression can be written in terms of $\delta\varepsilon$, then the FoM gradient is fully specified.

|  | $\boldsymbol{E}\left( \boldsymbol{r}_{0} \right)^{\boldsymbol{*}}\cdot\delta\boldsymbol{E}\left( \boldsymbol{r}_{0} \right)=\varepsilon_{0}\int_{\boldsymbol{r}^{\boldsymbol{'}}\mathcal{\in D}} \delta\varepsilon\left( \boldsymbol{r}^{'} \right)\boldsymbol{E}\left( \boldsymbol{r}_{0} \right)^{\boldsymbol{*}}\boldsymbol{\cdot}G\left( \boldsymbol{r}_{0},\boldsymbol{r}^{'} \right)\cdot\boldsymbol{E}\left( \boldsymbol{r}^{'} \right)d\boldsymbol{r}^{'}=\varepsilon_{0}\int_{\boldsymbol{r}^{\boldsymbol{'}}\mathcal{\in D}} \delta\varepsilon\left( \boldsymbol{r}^{'} \right)\boldsymbol{E(}\boldsymbol{r}^{'}\boldsymbol{)\cdot}G\left( {\boldsymbol{r}^{'}\boldsymbol{,r}}_{0} \right)\cdot\boldsymbol{E}\left( \boldsymbol{r}_{0} \right)^{*}d\boldsymbol{r}^{'}$ | (2.12) |
| --- | --- | --- |

The swapping of the fields around the Green’s function is enabled by a symmetry inherent in Maxwell’s equations which allows this operation to be done for electric dipoles in isotropic media. We can then define a new term:

|  | $\boldsymbol{E}_{\mathrm{adj}}\left( \boldsymbol{r} \right)\equiv G\left( \boldsymbol{r,r}_{0} \right)\cdot\boldsymbol{E}\left( \boldsymbol{r}_{0} \right)^{*}$ | (2.13) |
| --- | --- | --- |

This new expression is referred to as the “adjoint field” and is calculated as the field within the design domain that is induced by a dipole located at $\boldsymbol{r}_{0}$ driven by $\boldsymbol{E}\left( \boldsymbol{r}_{0} \right)^{*}$. We can now rewrite our FoMs in terms of the forward and adjoint fields calculated within the design domain. Examples of the forward and adjoint simulations performed to calculate forward and adjoint fields are shown below in Fig. S1.

|  | $\delta FoM_{I}=2\epsilon_{0}\mathrm{Re}\left\{ \int_{\boldsymbol{r}^{\boldsymbol{'}}\mathcal{\in D}} \delta\epsilon\left( \boldsymbol{r}^{'} \right)\boldsymbol{E}\left( \boldsymbol{r}^{'} \right)\cdot\boldsymbol{E}_{\mathrm{adj}}(\boldsymbol{r}^{'})d\boldsymbol{r}^{'} \right\}$ | (2.14) |
| --- | --- | --- |

|  | $\delta FoM_{\phi}=\left( \phi_{\mathrm{tgt}}-\phi\right)\frac{2\epsilon_{0}}{\left\vert E \right\vert^{2}}\mathrm{Im}\left\{ \int_{\boldsymbol{r}^{\boldsymbol{'}}\mathcal{\in D}} \delta\epsilon\left( \boldsymbol{r}^{'} \right)\boldsymbol{E}\left( \boldsymbol{r}^{'} \right)\cdot\boldsymbol{E}_{\mathrm{adj}}(\boldsymbol{r}^{'})d\boldsymbol{r}^{'} \right\}$ | (2.15) |
| --- | --- | --- |
|  |  |  |

Part 3: Fabrication Details

**Preparation of Crystalline Silicon Films on Pyrex Wafers**

1. A solution of hydrogen silsesquioxane in the form of Dow Corning XR-1541-006 was spun at 1500 rpm onto a 4” Pyrex wafer.
2. The Pyrex wafer was baked at 110ºC, 155ºC, and 210ºC for 2 minutes each.
3. A 4” silicon-on-insulator (SOI) wafer consisting of 250 nm of crystalline silicon on top of 3 μm of buried oxide on top of a silicon handle wafer was baked at 210ºC for 4 minutes.
4. The Pyrex wafer was bonded to an SOI wafer at 400ºC under high pressure.
5. The silicon handle wafer was mechanically ground from a thickness of 650 μm down to 100 μm.
6. A 12 mm x 18 mm grid of 3mm-wide polyimide tape was adhered to the back of the handle wafer.
7. The silicon handle wafer was etched away using an SF_6_ plasma.
8. The buried oxide layer was etched away using a 6% buffered HF solution, and the polyimide tape was removed.
9. The wafer was coated with a 10 μm layer of SPR 220-7 photoresist and diced into 12 mm x 18 mm pieces.
10. The photoresist was stripped using piranha solution, and the pieces subsequently cleaned in RCA SC1 and SC2 standard cleaning solutions.

**Production of Metasurfaces on Crystalline Silicon**

1. 4% AR-P 6200 positive electron-beam resist was spun onto a piece at 1500 rpm.
2. The piece was baked at 150 ºC for 90 seconds.
3. The piece was exposed using a 100 keV electron beam lithography machine.
4. The piece was developed in xylenes.
5. The piece de-scummed using an O_2_ plasma.
6. A 2 nm Ti adhesion layer along with 30 nm of Al_2_O_3_ were evaporated onto the piece to serve as a hard mask.
7. The piece was soaked in Microposit^TM^ remover 1165 overnight and sonicated to remove the electron-beam resist.
8. The patterns were etched into the piece using a Cl_2_/HBr plasma.
9. The remaining Ti and Al_2_O_3_ were removed by soaking the piece in 18% HCl at 50ºC.

Part 4: Experimental Setup and Characterization Details

**Experimental Setup**

A schematic of the optical setup used to characterize our metasurface lenses is shown in Fig. S2. A collimated beam of light from a tunable white light laser is filtered with a longpass filter and polarized with a linear polarizer. The beam then passes through a weak cylindrical lens in order to focus it onto the metasurface aperture without significantly changing the angle of incidence. The metasurface then focuses the light. The focal plane is then imaged onto a CMOS detector using a 0.9 NA, 100x objective and a tube lens.

**Calculating Efficiency**

An aperture is defined for calculating efficiency. For the devices with NAs of 0.2, it is a 10um wide slit; for the higher NA devices, it is a 5um wide slit. An image of the focal plane is acquired with a pixel size of approximately 72 nm. This image appears as a vertical line of focused light; however, a singular slice of pixels from the center is sampled for characterizing efficiency. Additionally, an image of a glass window with the same width as the lens and is taken, and a strip of pixels from the same location used in the focal plane image is isolated, cut off at the edges of the window.

Un-normalized relative efficiency: The un-normalized experimental relative efficiency is evaluated as the ratio of pixel intensities in the pre-defined aperture to the total intensities across the sampled line.

Un-normalized absolute efficiency: The un-normalized experimental absolute efficiency is evaluated as the ratio of pixel intensities in the aperture to the total intensities along the sampled line in the window image, taking into account the fact that the incident light on the devices does not include reflection from the glass-air interface on the window. For the normalized efficiencies, these values are divided by the efficiency of a theoretically perfect lens.

A perfect lens, of course, still creates a focus with fringes, and so will not be able to transmit 100% of incident power through an aperture. For each wavelength, aperture size, and numerical aperture, the theoretical maximum efficiency is calculated and used to normalize the experimental efficiencies. A 100% efficiency thus corresponds to an ideal lens.

Part 5: Linearization of 3D Metasurfaces

Just as we have used line segments to approximate a curve, so too can we use planar tiles to approximate a curved surface.

Consider a second-order surface $z_{1}\left( x,y \right)$ and a plane $z_{2}\left( x,y \right)$ used to approximate it:

|  | $z_{1}\left( x,y \right)=a_{1}x^{2}+a_{2}xy+a_{3}y^{2}+b_{1}x+b_{2}y+c_{1}$ | (5.1) |
| --- | --- | --- |

|  | $z_{2}\left( x,y \right)=b_{1}x+b_{2}y+c_{2}$ | (5.2) |
| --- | --- | --- |

The difference $\epsilon\left( x,y \right)$ between the two functions is:

|  | $\epsilon\left( x,y \right)=z_{1}\left( x,y \right)-z_{2}\left( x,y \right)=a_{1}x^{2}+a_{2}xy+a_{3}y^{2}+c_{3}$ | (5.3) |
| --- | --- | --- |

Where $c_{3}=c_{1}-c_{2}$. Over a square of side length $d$ centered around 0, the mean square value of the difference is:

$$\epsilon_{ms}\left( \pm d/2 \right)=\frac{1}{d^{2}}\int_{-d/2}^{d/2} \int_{-d/2}^{d/2} \left( a_{1}x^{2}+a_{2}xy+a_{3}y^{2}+c_{3} \right)^{2}dydx$$

$$=\frac{4}{d^{2}}\int_{0}^{d/2} \int_{0}^{d/2} \left[ a_{1}^{2}x^{4}+a_{3}^{2}y^{4}+2a_{2}\left( a_{1}x^{3}y+a_{3}xy^{3} \right)+\left( 2a_{1}a_{3}+a^{2} \right)x^{2}y^{2}+2c_{3}\left( a_{1}x^{2}+a_{3}y^{2} \right)+2a_{2}c_{3}xy+c_{3}^{2} \right]dydx$$

$$=\frac{4}{d^{2}}\left[ \left( a_{1}^{2}+a_{3}^{2} \right)\left( \frac{d^{5}}{5\times32} \right)\left( \frac{d}{2} \right)+2a_{2}\left( a_{1}+a_{3} \right)\left( \frac{d^{4}}{4\times15} \right)\left( \frac{d^{2}}{2\times4} \right)+\left( 2a_{1}a_{3}+a_{2}^{2} \right)\left( \frac{d^{3}}{3\times8} \right)\left( \frac{d^{3}}{3\times8} \right)+2c_{3}\left( a_{1}+a_{3} \right)\left( \frac{d^{3}}{3\times8} \right)\left( \frac{d}{2} \right)+2a_{2}c_{3}\left( \frac{d^{2}}{2\times4} \right)\left( \frac{d^{2}}{2\times4} \right)+c_{3}^{2}\left( \frac{d}{2} \right)\left( \frac{d}{2} \right) \right]$$

$$=\left( a_{1}^{2}+a_{3}^{2} \right)\frac{d^{4}}{80}+2a_{2}\left( a_{1}+a_{3} \right)\frac{d^{4}}{128}+\left( 2a_{1}a_{3}+a_{2}^{2} \right)\frac{d^{4}}{144}+2c_{3}\left( a_{1}+a_{3} \right)\frac{d^{2}}{12}+2a_{2}c_{3}\frac{d^{2}}{16}+c_{3}^{2}$$

|  | $\epsilon_{ms}=c_{3}^{2}+\left( \frac{a_{1}+a_{3}}{6}+\frac{a_{2}}{8} \right)d^{2}c_{3}+d^{4}\left( \frac{a_{1}^{2}+a_{3}^{2}}{80}+\frac{a_{2}\left( a_{1}+a_{3} \right)}{64}+\frac{a_{1}a_{3}}{72}+\frac{a_{2}^{2}}{144} \right)$ | (5.4) |
| --- | --- | --- |

The mean square error is minimized when its derivative with respect to the constant offset $c_{3}$ is zero:

|  | $\frac{d\epsilon_{ms}}{dc_{3}}=2c_{3}+\left( \frac{a_{1}+a_{3}}{6}+\frac{a_{2}}{8} \right)d^{2}=0$ | (5.5) |
| --- | --- | --- |

Therefore,

|  | $c_{3}=-\frac{d^{2}}{4}\left( \frac{a_{1}+a_{3}}{3}+\frac{a_{2}}{4} \right)$ | (5.6) |
| --- | --- | --- |

Substituting this value, we can explicitly calculate the mean square error as a function of $d$.

$$\epsilon_{ms}=-\frac{d^{4}}{16}\left( \frac{a_{1}+a_{3}}{3}+\frac{a_{2}}{4} \right)^{2}+\frac{d^{4}}{8}\left( \frac{a_{1}^{2}+a_{3}^{2}}{10}+\frac{a_{2}\left( a_{1}+a_{3} \right)}{8}+\frac{a_{1}a_{3}}{9}+\frac{a_{2}^{2}}{18} \right)$$

$$=\frac{d^{4}}{16}\left( \frac{a_{1}^{2}+a_{3}^{2}}{5}+\frac{a_{2}\left( a_{1}+a_{3} \right)}{4}+\frac{2a_{1}a_{3}}{9}+\frac{a_{2}^{2}}{9}-\frac{a_{1}^{2}+a_{3}^{2}}{9}-\frac{2a_{1}a_{3}}{9}-\frac{a_{2}^{2}}{16}-\frac{a_{2}\left( a_{1}+a_{3} \right)}{6} \right)$$

|  | $=\frac{d^{4}}{16}\left( \frac{4}{45}\left( a_{1}^{3}+a_{3}^{2} \right)+\frac{1}{12}a_{2}\left( a_{1}+a_{3}+\frac{7}{12}a_{2} \right) \right)$ | (5.7) |
| --- | --- | --- |

Therefore, the root-mean-square difference is:

|  | $\epsilon_{rms}=\frac{d^{2}}{4}\sqrt{\frac{4}{45}\left( a_{1}^{3}+a_{3}^{2} \right)+\frac{1}{12}a_{2}\left( a_{1}+a_{3}+\frac{7}{12}a_{2} \right)}$ | (5.8) |
| --- | --- | --- |
|  |  |  |

If for a particular function the cross term $a_{2}=0$, then we get a simplified expression similar to what we had found in the case of a line approximating a parabola.

|  | $\epsilon_{rms}=\frac{d^{2}\sqrt{a_{1}^{2}+a_{3}^{2}}}{6\sqrt{5}}$ | (5.8) |
| --- | --- | --- |

We can calculate the span in relation to a desired difference:

|  | $d=\sqrt{\frac{6\sqrt{5}}{\sqrt{a_{1}^{2}+a_{3}^{2}}}\epsilon_{rms}}$ | (5.9) |
| --- | --- | --- |

The Taylor series expansion for a surface is:

|  | $\phi\left( x,y \right)\approx\phi\left( x_{0},y_{0} \right)+\phi_{x}\left( x-x_{0} \right)+\phi_{y}\left( y-y_{0} \right)+\frac{1}{2}\left( \phi_{xx}\left( x-x_{0} \right)^{2}+\phi_{yy}\left( y-y_{0} \right)^{2}+2\phi_{xy}\left( x-x_{0} \right)\left( y-y_{0} \right) \right)$ | (5.10) |
| --- | --- | --- |

Therefore, the previously-determined quadratic constants are $a_{1}=0.5\phi_{xx}, a_{3}=0.5\phi_{yy}, a_{2}=\phi_{xy}$.

In a design example, we shall consider a lens. An ideal focusing lens with a focal length $f$ at a wavelength $\lambda$ has a phase profile of:

|  | $\phi\left( x,y \right)=\frac{2\pi}{\lambda}\left( f-\sqrt{f^{2}+x^{2}+y^{2}} \right)$ | (5.11) |
| --- | --- | --- |

Its derivatives are:

|  | $\phi_{x}\left( x,y \right)=-\frac{2\pi}{\lambda}\frac{x}{\sqrt{f^{2}+x^{2}+y^{2}}}$  $\phi_{y}\left( x,y \right)=-\frac{2\pi}{\lambda} \frac{y}{\sqrt{f^{2}+x^{2}+y^{2}}}$ | (5.12) |
| --- | --- | --- |

|  | $\phi_{xx}\left( x,y \right)=\phi_{yy}(x,y)=-\frac{2\pi}{\lambda}\frac{f^{2}}{{\sqrt{f^{2}+x^{2}+y^{2}}}^{3}}$ | (5.13) |
| --- | --- | --- |

|  | $\phi_{xy}\left( x,y \right)=-\frac{2\pi}{\lambda}\frac{xy}{{\sqrt{f^{2}+x^{2}+y^{2}}}^{3}}$ | (5.14) |
| --- | --- | --- |

The maximum curvature of the lens is in the center, where $x,y=0$. Here,

|  | $\phi_{xx}(0)=-\frac{2\pi}{f\lambda}$ | (5.15) |
| --- | --- | --- |

Since at this point $\phi_{xy}=0$, we can into (9) and find that the span $d$ and phase error $\epsilon_{rms}$ are related by:

|  | $d=\sqrt{\frac{12\sqrt{5}}{\sqrt{2}}\frac{\epsilon_{rms}}{2\pi}f\lambda}$ | (5.16) |
| --- | --- | --- |

For a maximum $\lambda/50$ RMS phase error, we let $\epsilon_{rms}<2\pi/50$, resulting in the constraint:

|  | $d<0.61\sqrt{f\lambda}$ | (5.17) |
| --- | --- | --- |

Equation 5.17 follows the same scaling law as Equation 1.16, but shows that the maximum linearization distance is somewhat smaller.

Part 6: Discussion on Optimization and Scaling of 3D Metasurfaces

Suppose we wish to optimize a large-area metasurface in the shape of a square with side length $L$. Optimizing the entire device at once has a runtime of $O\left( L^{2.4}\times L^{2.4} \right)=O(L^{4.8})$, which is very slow. Alternatively, we can break the entire metasurface into smaller squares of side length $d$ and optimize each one, as shown in Fig. S4. The runtime is now $O\left( d^{4.8}\times\left( L/d \right)^{2} \right)\equiv O\left( L^{2} \right)$.

Although the stitching approach does provides a large runtime improvement, the polynomial runtime still does not scale well with size. Furthermore, the algorithms for optimizing 3D sections are much more complex than the algorithms for optimizing 2D structures as discussed in the manuscript. More research is required to improve the optimization algorithms so that they can be effectively applied to arbitrary 3D metasurfaces.

In the design of a lens, one can take advantage of symmetry to reduce the problem’s complexity. As shown in Fig. S5, a lens can be divided into concentric rings. Only one device needs to be optimized per ring, and it can be rotated and tiled around the ring. This reduces the runtime back down to $O\left( L \right)$, which is much more manageable.

This tiling scheme requires wedge-shaped sections, but most simulators and optimizers deal with rectangular devices. Thus, the boundaries between the sections will contribute an unknown amount of stitching error to the final device. This problem will become more exacerbated at the center where each ring’s radius of curvature is smaller. To mitigate this effect, one may use conventional methods to design the center of the lens and use optimization to design the outer rings.

Part 7: Outline and Flow Chart of Design Algorithm

Below is an outline of the design methodologies used in this manuscript. A flow chart of the design process is shown in Figure S6.

1. **Assessment of computational efficiency**: splitting one large problem into multiple smaller problems greatly reduces the order of complexity.
2. **Sectioning of a curvilinear phase profile**: the splitting method introduces wavefront error. This error is analyzed and guidelines are provided on how to minimize the error.
3. **Topology optimization for finite-sized, isolated devices**: electromagnetic devices can be optimized using the adjoint method, given a suitable simulation basis.
4. **Aperiodic Fourier Modal Method**: finite-sized aperiodic devices can be simulated by modifying a periodic simulator with coordinate transformations.
5. **Section optimization demonstration**: we demonstrate capabilities of combining the methods introduced above.

**Figures**


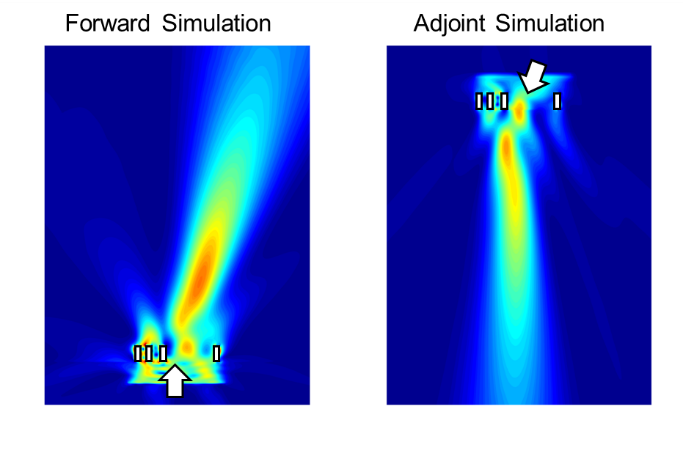


**Figure S1, Examples of forward and adjoint simulations on a binarized structure.**


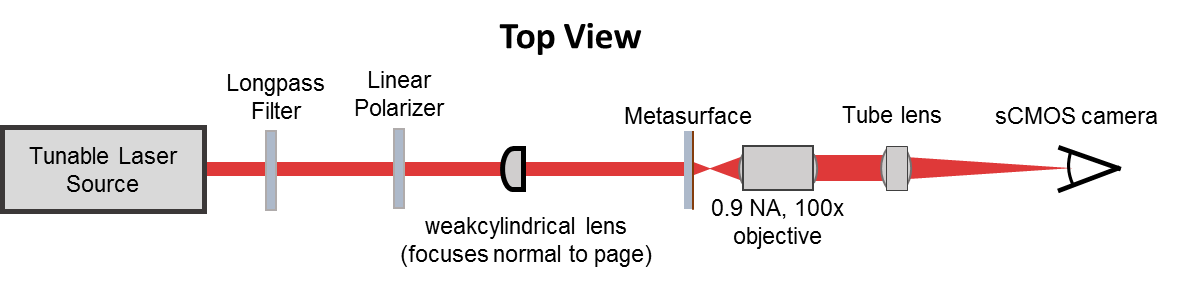


**Figure S2, Optical setup for characterizing metasurface lenses.**


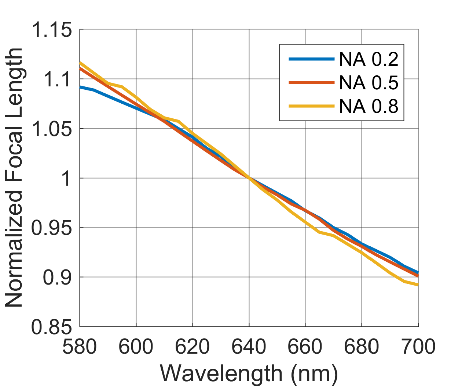


**Figure S3, Simulated focal length shifts of the metalenses as a function of wavelength.**

**
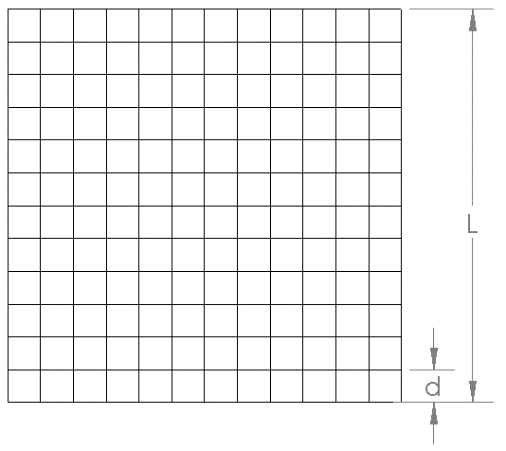
**

**Figure S4, Rectangular tiling scheme for arbitrary metasurfaces.**

**
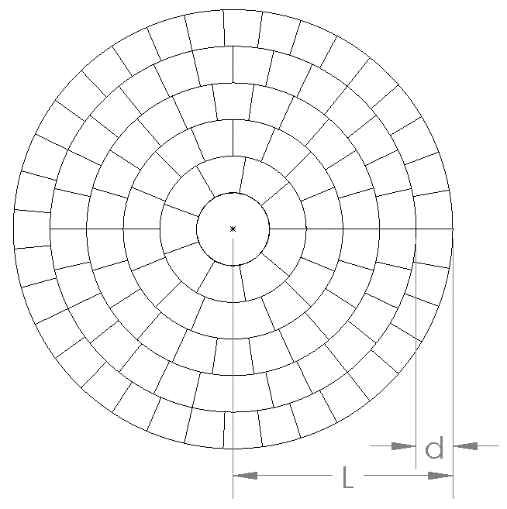
**

**Figure S5, Radial tiling scheme for metasurface lenses.**

**
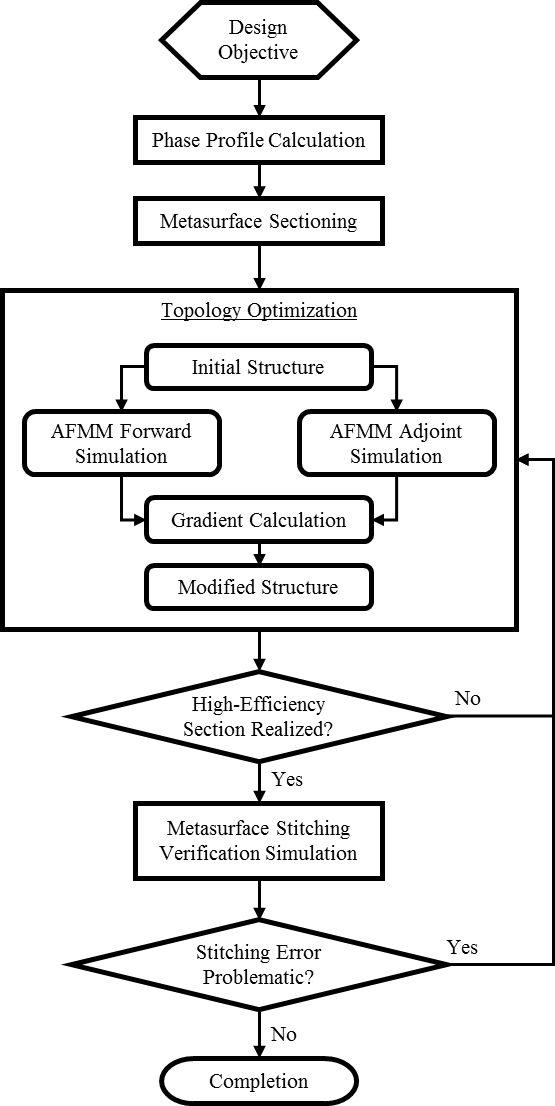
**

**Figure S6, Flow chart of metasurface design process.**
